# Genetic and epigenetic effects on couple adjustment in context of romantic relationship: A scoping systematic review

**DOI:** 10.3389/fgene.2023.1002048

**Published:** 2023-01-24

**Authors:** Pouria Khani, Mitra Ansari Dezfouli, Farzad Nasri, Maryam Rahemi, Salma Ahmadloo, Hamed Afkhami, Farzane Saeidi, Sergey Tereshchenko, Mohammad Reza Bigdeli, Mohammad Hossein Modarressi

**Affiliations:** ^1^ Department of Medical Genetics, School of Medicine, Tehran University of Medical Sciences (TUMS), Tehran, Iran; ^2^ Department of Neuroscience, School of Advanced Technologies in Medicine, Tehran University of Medical Sciences (TUMS), Tehran, Iran; ^3^ Immunology Research Center, Iran University of Medical Sciences, Tehran, Iran; ^4^ Department of Immunology, School of Medicine, Iran University of Medical Sciences, Tehran, Iran; ^5^ Department of stem cell technology and tissue regeneration, Faculty of Science, Tehran University, Tehran, Iran; ^6^ Faculty of Life Sciences and Biotechnology, Shahid Beheshti University, Tehran, Iran; ^7^ Department of Medical Microbiology, Faculty of Medicine, Shahed University of Medical Sciences, Tehran, Iran; ^8^ Department of Medical Genetics, School of Medical Sciences, Tarbiat Modares University, Tehran, Iran; ^9^ Research Institute of Medical Problems of the North, Federal Research Center “Krasnoyarsk Science Center of the Siberian Branch of the Russian Academy of Sciences”, Krasnoyarsk, Russia; ^10^ Institute for Cognitive and Brain Sciences, Shahid Beheshti University, Tehran, Iran

**Keywords:** genetics, epigenetics, romantic relationship, couple adjustment, marital adjustment

## Abstract

**Introduction:** Couples’ relationships defined by a complex interaction between the two partners and their intrapersonal traits. Romantic; relationships and love are associated with marital satisfaction and stability, as well as couples’ happiness and health. Personality traits influence romantic relationships and, personality influenced by genetical and non-genetically factors. The roles of non-genetically factors such as socioeconomic position and external appearance have revealed in determining the quality of romantic relationships.

**Methods:** We; performed a scoping systematic review to assess the association between genetics and epigenetic factors and romantic relationship. Relevant articles were identified by PubMed, EMBASE, Web of Science, Scopus, and the APA PsycInfo searching between inception and 4 June 2022.

**Results:** Different studies evaluated the associated polymorphisms in 15 different genes or chromosomal regions. In the first step; we classified them into four groups: (1) Oxytocin-related signaling pathway (*OXTR*, *CD38*, and *AVPR1A*); (2) Serotonin-related signaling pathway (*SLC6A4*, *HTR1A*, and *HTR2A*); (3) Dopamine and catecholamine-related signaling pathway (*DRD1*, *DRD2*, *DRD4*, *ANKK1*, and *COMT*); and (4) other genes (*HLA*, *GABRA2*, *OPRM1*, and Y-DNA haplogroup D-M55). Then, we evaluated and extracted significant polymorphisms that affect couple adjustment and romantic relationships.

**Discussion:** Overall, the findings suggest that genetic and epigenetics variants play a key role in marital adjustment and romantic relationships over time.

## Introduction

Personality neuroscience as a research discipline has focused on understanding individual differences in such important psychological areas of motivation, emotion, cognition, and behavior (Braver et al., 2014). An essential part of personality neuroscience seeks to find the molecular genetics behind individual differences. Based on the “first law” of behavior genetics science, all human behavioral traits are heritable (Chabris et al., 2015). Genetic influences on personality differences are ubiquitous, but their nature is not well-understood (Penke et al., 2007). By adopting this approach and law, researchers in different fields, such as genetics, psychology, sociology, politics, and other sciences, have attempted to find a link between behaviors and specific genes.

Couple adjustment is a process that expresses the degree of satisfaction between couples and their level of cohesion, consensus, and troublesome differences. It also reflects interpersonal tensions and anxiety. Studies have found that adjusted-happy couples experience higher levels of sexual satisfaction and less distress in their marriages. Distress in marriage is linked to an increased risk for mental and physical health problems, including depression and anxiety (Spanier, 1976; Fisher et al., 2015; Stokes, 2017; Whisman et al., 2018). Fletcher, G. J. and their colleagues proposed that pair bonding is a coupling formation in which males and females live together for a relatively lasting time. This manner is related to monogamous mating arrangement. They claimed that romantic love is an essential fundamental motivating energy supporting monogamy and long-term couple bonding in humans (Fletcher et al., 2015). The formation of romantic attachment is a developmental process with changes over time, such as a slow consolidation of intimacy between partners as the relationship progresses (Aron et al., 2005). Attachment is a theoretical framework that includes aspects of a person’s life throughout his/her life. From the point of view of the scientists studying mental health, this theory is considered a beneficial model for analyzing close relationships and individual differences in the regulation of emotions. According to attachment theory, the desire to establish close emotional relationships with specific people, called “attachment figures,” is an important part of human nature that exists from infancy and is observed consistently throughout a person’s life. This theory suggests that the desire of humans to create stable emotional bonds and maintain them is innate. The attachment term in this theory referred to the emotional, cognitive, and behavioral processes involved in the formation and maintenance of relationships (Bowlby, 1973; Bowlby, 1982; Sroufe, 1986; Rholes and Simpson, 2004).

Romantic relationships are characterized by a specific intensity, precise expressions of affection, and initiation into sexual encounters (Collins et al., 2009). Romantic love is correlated with marital satisfaction and stability and couples’ happiness and health. Individuals who are involved in long-term love partnerships revel in healthier and longer lives (Hatfield et al., 2008; Diamond et al., 2010). Couples’ relationships are defined by an intricate interaction between partners and their intrapersonal traits (Lazaridès et al., 2010). Marital disruption is an acute life stressor. Divorce represents a gradual process that encompasses affective, cognitive, behavioral, social, and socioeconomic changes from marriage to divorce (Zeigler-Hill and Shackelford, 2020). Genetic components affect the chance of divorce, which can be passed from generation to generation (McGue and Lykken, 1992; Salvatore et al., 2018). One of the determinants of the quality of marriage is personality.

Personality traits affect romantic relationships. From a scientific point of view, personality is defined as particular and specific patterns of thinking, feeling, and behavior in a person (Roberts, 2009). Individual personality differences are often measured by using the five-factor model (FFM), also known as the “Big Five.” It uses five broad dimensions (extroversion, agreeableness, conscientiousness, neuroticism, and openness) to model personality (Judge et al., 1999; Cobb-Clark and Schurer, 2012). Different studies offer robust proof of human personality heritability. Many genes are expected to influence the heritability and development of personality in concert, rather than separately (Judge et al., 1999; Zwir et al., 2020). Understanding romantic relationship outcomes has improved due to research examining gene–environment correlations and gene-by-environment interactions (Whisman and South, 2017). GWAS (genome-wide association studies) revealed a significant association between SNPs or chromosomal locations and the FFM. For example, more excellent scores of neuroticism, extraversion, and agreeableness are identified in the 5q34–q35, 3p24, and 3q13 regions (Luciano et al., 2012; Kim et al., 2013; De Moor et al., 2015). New tools such as next-generation sequencing allow us to search and analyze connections between genes and personality traits (Zmorzyński et al., 2021).

In this systematic review, we argue about genetic and epigenetic factors that can influence couple adjustment and romantic relationships over time.

## Materials and methods

### Search strategy

**FIGURE 1:**
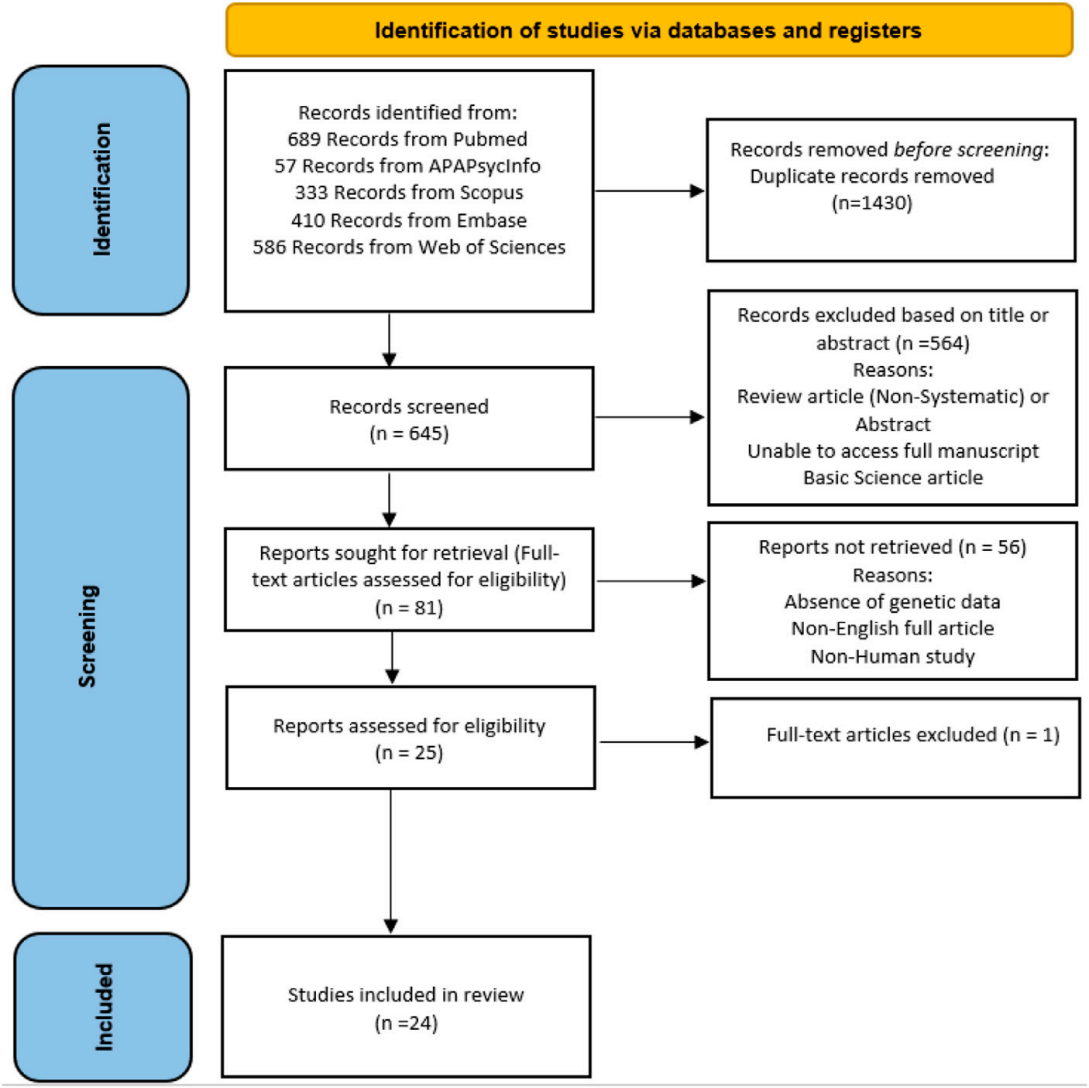
PRISMA flow diagram of study identification.

We followed the Preferred Ideal Reporting Items for Systematic Review and Meta-Analyses (PRISMA) indications in the study indication and selection (Figure 1) (Moher et al., 2009). Relevant article identification for this study was performed by searches of PubMed, EMBASE, Web of Science, Scopus, and the APA PsycInfo register between inception and 4 June 2022. The PubMed search was as follows: (genetics [title/abstract] OR polymorphism [title/abstract] OR genome-wide [title/abstract] OR genome-wide [title/abstract] OR Epigenetics [title/abstract]) AND (“romantic relationship” [title/abstract] OR “romantic love” [title/abstract]) and (genetics [title/abstract] OR polymorphism [title/abstract] OR genome-wide [title/abstract] OR genome-wide [title/abstract] OR Epigenetics [title/abstract]) AND (“couple adjustment” [title/abstract] OR “couple attachment” [title/abstract] OR “marital adjustment”). This was adapted according to each database’s needs. We also studied the reference lists of original reports and reviews. Place (the country) of research was not a limiting factor in this search strategy, and papers published in English were reviewed.

### Inclusion and exclusion criteria

We included studies that meet the following criteria: 1) molecular and genetic basis of couple adjustment and its impact on the romantic relationship or romantic love and 2) full articles published in English. In this study, we excluded 1) the studies that were solely related to psychological aspects of a romantic relationship, 2) studies that were reviews or abstracts, and 3) studies whose information was inaccessible.

### Data collection and synthesis

Three authors (PKH, MR, and FN) independently reviewed and extracted the data from identified eligible studies, and disagreements were resolved by consulting a third author (ST, SA, or FS). The following data were extracted: the first author’s name, year of publication, genes inspected, experimental techniques used, and sample size (Table 1). We classified the findings of these studies according to the following subjects (Table 2): 1) the oxytocin-related signaling pathway; 2) the serotonin-related signaling pathway; 3) the dopamine- and catecholamine-related signaling pathway; and 4) other genes. We did not carry out a meta-analysis because we were not able to classify studies based on their appropriate similarity in study design and experimental techniques used, genetic variants that were examined, or the manner in which the results were presented. Finally, we used the Newcastle–Ottawa scale (NOS) to assess the methodological quality of the included studies. The universities of Newcastle, Australia, and Ottawa, Canada, collaborate on the Newcastle–Ottawa scale (NOS) on a regular basis. It was created with the goal of assessing the quality of non-randomized studies, with its design, content, and ease of use directed toward incorporating the quality assessments in the interpretation of meta-analytic results. A “star system” has been developed in which studies are judged based on three broad perspectives: study group selection, group comparability, and ascertainment of either the exposure or outcome of interest for case-control or cohort studies. This project’s objective is to create a tool that will make it simple and practical to evaluate the quality of non-randomized studies for inclusion in a systematic review (Wells et al., 2000).

**TABLE 1:** Features of the included studies.

First author	Year	Gene	Evaluated polymorphism(s)	Studied population/ancestry	Experimental technique	Sample size	NOS	References
Masahiro Matsunaga	2021	Y-chromosomal (Y-DNA) haplogroup D ancestor	Y-DNA haplogroup D-M55	Japanese	Taqman SNP genotyping assays	623	8	Matsunaga et al. (2021)
Anastasia Makhanova	2021	*CD38*	rs3796863	United States (White/Caucasian)	Taqman SNP genotyping assay	142	8	Makhanova et al. (2021)
Gentiana Sadikaj	2020	*CD38*	rs3796863	Canadian	PCR	222	8	Sadikaj et al. (2020)
Bianca P. Acevedo	2020	*AVPR1A*, *OXTR*, *COMT*, and *DRD4*	AVPR1a rs3, rs53576, rs4680, and DRD4-7R	United States (California, New York)	Fragment analysis and MassARRAY Compact system	19	7	Acevedo et al. (2020)
Joan K. Monin	2019	*OXTR*	rs53576	United States (New Haven)	Taqman SNP genotyping assays	356	8	Monin et al. (2019)
Steven M. Kogan	2019	*OXTR*	OXTR methylation	African–American men (resided in 11 rural counties in South Georgia)	Bisulfite	309	7	Kogan et al. (2019)
Kristina Tchalova	2019	*OPRM1*	C77G in primates and A118G in humans	Canada (Montreal)	Sequencing	184	8	Tchalova et al. (2021)
Eiluned Pearce	2018	*OXTR*, *AVPR1A*, *OPRM1*, *AR*, *DRD1*, *DRD2*, *ANKK1*, *HTR1A*, and *HTR2A*	10 OXTR SNPs, two AVPR1A SNPs, five OPRM1 SNPs, one AR SNP, 1 DRD1 SNP, two DRD2 SNPs, one ANKK1 SNP, one HTR1A SNP, and one HTR2A SNP	Three types of samples: a healthy Caucasian sample, a subclinical sample (Caucasian individuals with histories of mental illness), and a non-White sample (four Black African, 12 Chinese, 20 Indian subcontinent, six other Asian, 16 mixed Black Caribbean, and eight others)	NA	206	8	Pearce et al. (2018a)
Eiluned Pearce	2018	*AR, OXTR*, *AVPR1A*, *OPRM1*, *DRD1/2*, *ANKK1*, and *5HTR1A/2A*	Associations between 2D:4D and single-nucleotide polymorphisms (SNPs) in nine neurochemical receptor genes	Caucasians with no history of psychopathology	NA	474	8	Pearce et al. (2018b)
Eiluned Pearce	2017	*OXTR*, *AVPR1a*, *OPRM1*, *DRD1*, *DRD2*, *ANKK1*, *HTR1A*, *HTR2A*, and *AR*	33 candidate SNPs from nine genes: 11 candidate SNPs for OXTR (oxytocin), three SNPs for AVPR1a (vasopressin), six SNPs for OPRM1 (β-endorphins), three SNPs for DRD1, three for DRD2, one SNP for ANKK1 (located downstream from DRD2) (dopamine), one SNP for HTR1A, two SNPs for HTR2A (serotonin), and one SNP for AR (testosterone)	Caucasians with no history of psychopathology	NA	757	8	Pearce et al. (2017)
Man-Kit Lei	2017	*SLC6A4*	5-HTTLPR	African–American families	PCR	270	8	Lei et al. (2016)
Ronald L. Simons	2017	*OXTR*	OXTR methylation	African–American women	Illumina 450 K Human Methylation Beadchip	100	8	Simons et al. (2017)
J. Kromer	2016	*HLA*	HLA class I/II	German	Next-generation sequencing	508	8	Kromer et al. (2016)
Lisa R. Starr	2016	*SLC6A4*	5-HTTLPR	Australia (Caucasian ancestry and other racial groups (Asian–Australian, Maori/Islander, and Australian Aborigine)	PCR	381	7	Starr and Hammen (2016)
Siyang Luo	2015	*SLC6A4*	5-HTTLPR	Chinese	PCR	1,532	8	Luo et al. (2016)
Sara B. Algoe	2014	*CD38*	rs6449182 and rs3796863	United States (North Carolina)	Taqman SNP genotyping assays	128	8	Algoe and Way (2014)
Jinting Liu	2014	*5-HT1A*	rs6295	Chinese	PCR	579	8	Liu et al. (2014)
Pingyuan Gong	2014	*5-HT1A*	rs6295	Chinese	PCR-SSCP	504	8	Gong et al. (2014)
April S. Masarik and Rand D. Conger	2014	*5-HTT (SLC6A4)*, *ANKK1*, *DRD2*, *DRD4*, and *COMT*	5-HTTLPR, A1 allele of the Taq1A polymorphism in ANKK1/DRD2, 7R allele of exon 3 VNTR in DRD4, and Met allele of the Vall58Met polymorphism in *COMT*	—	PCR, Taqman real-time PCR	352	8	Masarik et al. (2014)
Inna Schneiderman	2014	*OXTR*	rs13316193, rs2254298, rs1042778, rs2268494, and rs2268490	Caucasians, who are healthy and completed at least 12 years of education	SNaPshot method and High Resolution Melt (HRM)	120	8	Schneiderman et al. (2014)
Claudia M. Haase	2013	*SLC6A4*	Serotonin transporter promoter polymorphism (5-HTTLPR)	United States (San Francisco Bay Area)	PCR	125	8	Haase et al. (2013)
Ronald L. Simons	2013	*GABRA2*	Block 1: rs531460, rs567926, and rs279858; block 2: rs1440130 and rs279837	African–American	Taqman MGB assays	549	8	Simons et al. (2013)
Hasse Walum	2012	*OXTR*	rs75775, rs1488467, rs4564970, rs53576, rs237897, rs237887, rs11720238, rs4686302, rs2254298, rs2268493, rs1042778, and rs7632287	Sweden	KBioscience using the KASPar chemistry, Taqman SNP genotyping assays	1,240	8	Walum et al. (2012)
Hasse Walum	2008	*AVPR1A*	GT25 repeat polymorphism, RS3 repeat polymorphism, and RS1 repeat polymorphism	Sweden	Fragment analysis	1,899	8	Walum et al. (2008)
Garver-Apgar CE	2006	MHC	MHC alleles at the A, B, and DRB loci	New Mexico	Allele-specific primers to amplify PCR products	48	7	Garver-Apgar et al. (2006)

NOS, Newcastle–Ottawa scale; NA, not available.

**TABLE 2:** Different evaluated genes or chromosomal regions.

Signaling pathway	Gene	Description	Location
Oxytocin-related signaling pathway	*OXTR*	Oxytocin receptor	3p25.3
	*CD38*	CD38 molecule	4p15.32
	*AVPR1A*	Arginine vasopressin receptor 1A	12q14.2
Dopamine- and catecholamine-related signaling pathway	*DRD1*	Dopamine receptor D1	5q35.2
	*DRD2*	Dopamine receptor D2	11q23.2
	*DRD4*	Dopamine receptor D4	11p15.5
	*ANKK1*	Ankyrin repeat and kinase domain containing 1	11q23.2
	*COMT*	Catechol-O-methyltransferase	22q11.21
Serotonin-related signaling pathway	*SLC6A4* (*5-HTT*)	Solute carrier family 6 member 4	17q11.2
	*HTR1A* (*5-HT1A*) (*5-HTR1A*)	5-Hydroxytryptamine receptor 1A	5q12.3
	*HTR2A* (*5-HTR2A*)	5-Hydroxytryptamine receptor 2A	13q14.2
Other genes	*MHC*	Class I and II major histocompatibility complex	6p21
	*GABRA2*	Gamma-aminobutyric acid type A receptor subunit alpha-2	4p12
	*ORPM1*	Opioid receptor Mu 1	6q25.2
	Y-DNA	Y-DNA haplogroup D-M55	Y chromosome

## Results

**FIGURE 2:**
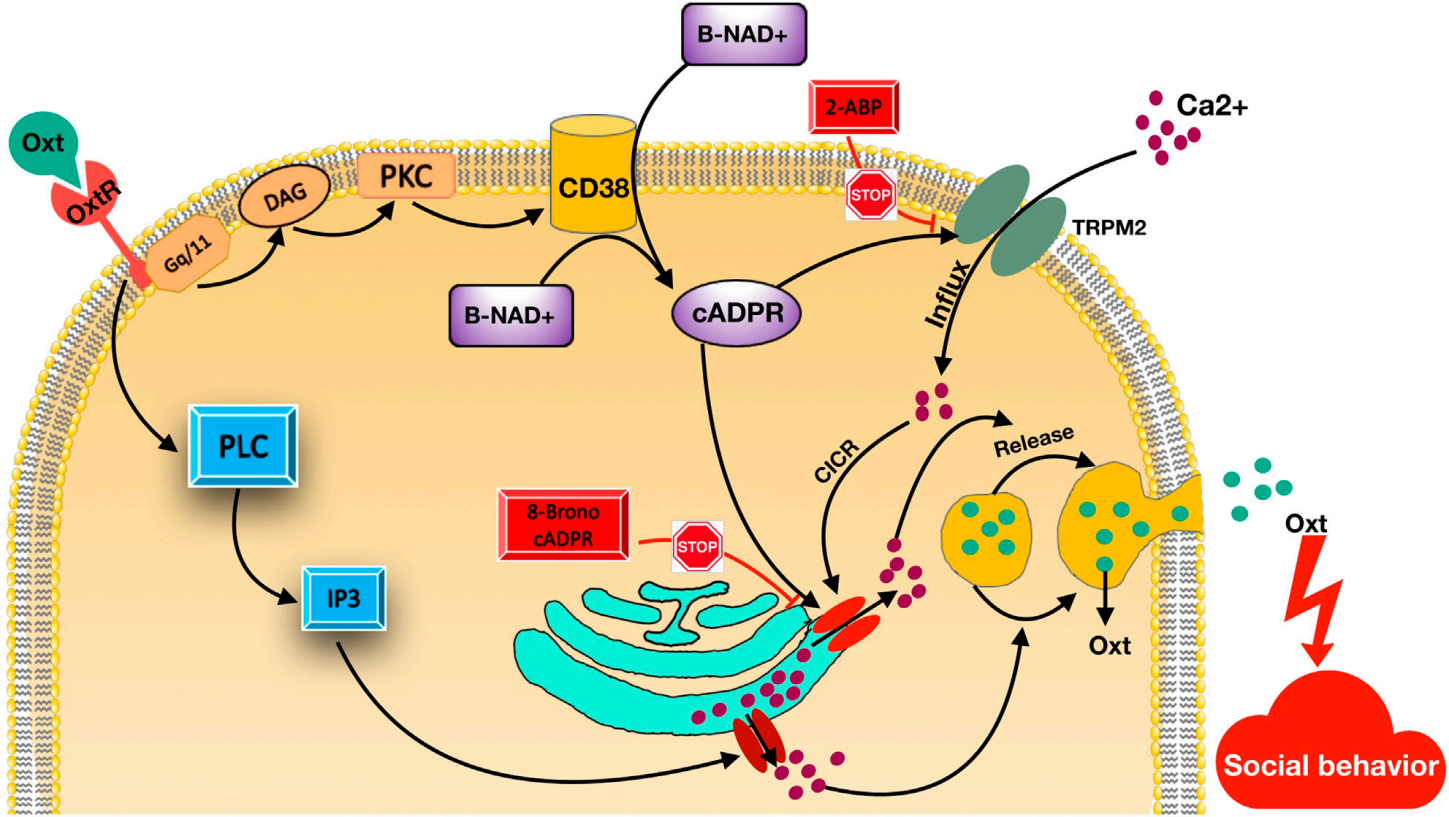
Oxytocin- related signaling pathway. Oxytocin receptors (OTR) stimulated by Oxytocin (OT; green circles). Then, the G_q/11_ type GTP-binding protein and phospholipase C (PLC) are activated, leading to creation of inositol-1,4,5-trisphosphate (IP3) and diacylglycerol (DAG). This results in Ca^2+^ mobilization activation from IP_3_-sensitive Ca^2+^ pools. CD38 activated by stimulated protein kinase C (PKC) and increases cADPR creation from β-NAD^+^ inside or outside cells. cADPR using a mechanism mentioned as Ca^2+^-induced Ca^2+^ release, mobilizes Ca^2+^ via cADPR-sensitive Ca^2+^ pools. cADPR triggers Ca^2+^ influx TRPM2 cation channels. TRPM2 channels can inhibit by 2-Aminoethoxydiphenyl borate (2-APB). TRPM2 facilitates Ca^2+^ influx, which also trigger Ca^2+^ mobilization through ryanodine receptor Ca^2+^ release channels as a cofactor together with cADPR. These Ca^2+^ intensification mechanisms increase Ca^2+^ ions and Ca^2+^ ions trigger OT release into the brain, which is an important factor for social memory and social behavior.

**FIGURE 3:**
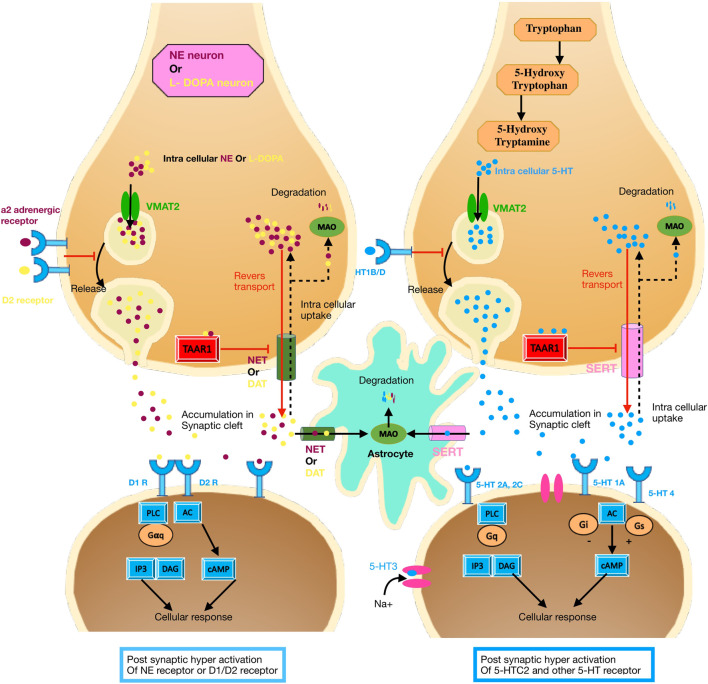
Serotonin-related signaling pathway; Dopamine and catecholamine-related signaling pathway. **(A)** Dopamine synthesis and signal transduction. The main metabolic pathway for dopamine synthesis has two steps. First, tyrosine hydroxylase can convert tyrosine to L-dopa and then L-dopa transforms to dopamine (not shown). Dopamine is transported by the monoamine transporter (VMAT2) from the cytosol to the vesicles in synaptic vesicles and is stored until it is released into the synaptic cleft. Dopamine degradation pathway involves monoamine oxidase (MAO) in the outer mitochondrial membrane. Dopamine receptors are present in both postsynaptic and presynaptic neurons (including dopamine transporters, DAT). Dopamine receptors belong to the GPCR superfamily associated with various types of G proteins. D1- and d2-like receptors are important receptors for dopamine signaling. These receptors are also crosstalks with other signaling pathways such as Gαq, Gβγ, DAG, IP3, CAMP, and MAPK-MEK-ERK. **(B)** 5-HT Synthesis and signal transduction. Tryptophan is the essential amino acid involved in the synthesis of 5-HT. In CNS l-tryptophan is hydroxylated to 5-hydroxytryptophan (5-HTP) by the enzyme tryptophan hydroxylase type 2 (TPH2). This is followed by subsequent decarboxylation THAT transforms 5-hydroxytryptophan into 5-hydroxytryptamine. 5-HT is transported by the monoamine transporter) VMAT2) into vesicles and storage. Like dopamine 5-HT can be degraded by monoamine oxidase (MAO) in the outer mitochondrial membrane. After released 5-HT it can engage with receptors. All 5-HTRs are heteroreceptors and postsynaptically expressed on non-serotonergic neurons and autoreceptors located presynaptically on the serotonergic neurons. 5-HT1A, B, D, E, F, 5-HT2A, B, C, 5-HT4, 5-HT5A, B, 5-HT6, and 5-HT7 receptors are classified as G protein-coupled receptors (GPCRs), while 5-HT3A, B, C, D, E receptors are ligand-gated ion channels. Upon ligand binding, the intracellular loop and C-terminal tail interact with specific G protein families, including Gαs, Gαi/o, Gαq/11, and leading to activated many signaling pathways such as DAG, IP3, CAMP, and MAPK-MEK-ERK.

A total of 2075 studies were found in the preliminary search. After a comprehensive assessment comprising the removal of duplicates, exclusion based on title or abstract, and exclusion based on certain reasons such as reachability to their data, 24 studies were finally included in this review. Different studies evaluated the associated polymorphisms in 16 diverse genes or chromosomal regions. After collecting a list of these genes, we classified them into four groups, namely, 1) the oxytocin-related signaling pathway Figure 2; 2) the serotonin-related signaling pathway Figure 3; (Penke et al., 2007) the dopamine- and catecholamine-related signaling pathway (Figure 3); and 4) other genes Table 2.

### Oxytocin-related signaling pathway

Oxytocin (OT) and arginine vasopressin (AVP) are typically produced in specific neurons in the hypothalamus in the supraoptic and paraventricular nuclei (Hoyle, 1998; Gainer, 2012). They have multiple physiological roles in peripheral organs such as the uterus and kidneys (Leng et al., 2015). These two hormones and their receptors are present in the brain both in women (pregnant and non-pregnant) and men (Leng and Russell, 2002; Brunton and Russell, 2010). Research has shown that in addition to classical hormonal functions, these hormones play an essential role in the processes of human social cognition and social behavior (Donaldson and Young, 2008).

The OT receptor is a seven-transmembrane protein coupled with the G_q/11_-type GTP-binding protein (Figure 2). OT receptor stimulation leads to inositol-1,4,5-trisphosphate (IP_3_) and diacylglycerol (DAG) production by the activation of phospholipase C (PLC) (Gimpl and Fahrenholz, 2001). This results in Ca^2+^ mobilization stimulation from IP_3_-sensitive Ca^2+^ pools (Lopatina et al., 2010). On the other hand, the cyclic ADP-ribose (cADPR) Ca^2+^ signaling pathway has been recognized downstream of OT receptors (Higashida et al., 2010; Lee, 2012). cADPR, using a mechanism mentioned as Ca^2+^-induced Ca^2+^ release, mobilizes Ca^2+^ *via* cADPR-sensitive Ca^2+^ pools. In this process, cADPR has a key role in mobilizing Ca^2+^-utilizing ryanodine receptors (Lambert et al., 1994; Fill and Copello, 2002; Endo, 2009). Several different ways are known for regulation of intracellular cADPR concentrations comprising ADP-ribosylcyclase or CD38 activation or the G protein-coupled receptor phosphorylation as downstream signaling pathways (Boittin et al., 2003; Sternfeld et al., 2003; Higashida et al., 2007). It has been suggested that internalization of produced cADPR in extracellular space *via* CD38 into fibroblasts and astrocytes leads to stimulation of intracellular ryanodine receptors (Franco et al., 2001; De Flora et al., 2004).

### Oxytocin receptor gene (*OXTR*)

The oxytocin (OT) pathway system is actively involved in socialization and regulation of interpersonal interaction (Donaldson and Young, 2008; Meyer-Lindenberg et al., 2011). Numerous studies have shown an association of OT with an increase in emotionally colored social contacts and increased trust in the communication process (Grewen et al., 2005; Kosfeld et al., 2005). Intranasal administration of OT resulted in a more remarkable ability to absorb social information and a greater expression of altruistic personality traits (Kosfeld et al., 2005; Rimmele et al., 2009; De Dreu et al., 2010). Because of the conservation of the neuropeptide oxytocin in different mammalian species and the inheritability of human sociality, variations in genes encoding oxytocin may explain personal differences in sociality (De Dreu et al., 2010; Meyer-Lindenberg et al., 2011). It has been suggested that the oxytocin receptor gene (*OXTR*) (on 3p25 ) could potentially be an important candidate for sociality behaviors (Rodrigues et al., 2009).

In recent years, the genetic aspects affecting the production and receptivity of OT have engrossed the attention of psychopathology researchers. The main focus of this research has been on the study of single-nucleotide polymorphisms of the *OXTR* gene (rs53576 and rs2254298); the *OXT* gene (rs2740210, rs4813627, and rs4813625); and the *CD38* gene (rs3796863 and rs6449197) (Feldman et al., 2016). Some genetic variants have been associated with increased aggressiveness (Shao et al., 2018), sociality (Parris et al., 2018), significant problems in interpersonal relationships (Andreou et al., 2018), and hyperactivity (Ayaz et al., 2015). A detailed review of the association of OT pathway genes is presented by Feldman et al. (2016) and Cataldo et al. (2018).

It has been found that *OXTR* rs53576 can play a role in pair bonding (Poulin et al., 2012; Acevedo et al., 2019). Studies have shown that people with more G alleles have higher levels of sociability, empathy, and altruism with their emotional partner (Uzefovsky et al., 2016; Gong et al., 2017). Acevedo et al. (2020) reported that some genetic polymorphisms, including *OXTR* rs53576, correlate with romantic love maintenance among first-time newlyweds. The simulated interaction model for *OXTR* rs53576 with sustaining romantic love appeared in the septum (bilaterally) and left VTA (L). Activation of L VTA is frequently found in studies on facial attractiveness, which revealed that L VTA specifically reacted to smiling and supportive faces (Vrtička et al., 2008). A similar study showed that men who received the OT hormone through intranasal administration experienced L VTA activation in response to seeing their partner’s image (Scheele et al., 2013). Various findings show that the effects created by the OT hormone are specifically related to the emotional partner, which can strengthen and improve attachment and pair bonding. The point to be made is that sex may influence the effects of OT on mate choice and pair bonding. Individual variances such as personality and adjustment style can affect the relationship of OT with couples’ bonding choices (Pearce et al., 2019; Xu et al., 2020). The other study reported greater marital satisfaction in individuals with the GG genotype in *OXTR* rs53576 (compared with AA or AG genotypes) (Monin et al., 2019).

However, a meta-analysis study in 2015 reported conflicting findings about the *OXTR* rs53576 variation (Li et al., 2015). This meta-analysis showed that homozygous individuals for the G allele mostly had increased social behavior than the carriers of the A allele. In addition, they did not find a significant difference in close relationship measures between homozygous individuals for the G allele and the carriers of the A allele. In summary, this study suggested that *OXTR* rs53576 can forecast how an individual reacts to other people, but it might not be linked to individual variances in forming a close relationship (i.e., parent–child or romantic/marital) (Li et al., 2015).

Pearce et al. (2017), in a preliminary study, showed that for dyadic relationships, two *OXTR* SNPs (rs2268490 and rs4686302) had significant effects on Relationship Assessment Scale (RAS) scores of relationship quality, whereas rs2254298 showed a trend toward significance. Also, seven *OXTR* SNPs significantly correlate with Sociosexual Orientation Inventory-Revised (SOI-R) scores: rs237887, rs2268490, rs2254298, rs13316193, rs53576, rs237897, and rs4686302. In terms of personal network size, there was a significant correlation with *OXTR* rs237887. For connection feelings to their local community, *OXTR* rs53576 showed a significant correlation (Pearce et al., 2017). In another replicate study, it was shown that *OXTR* rs237897, rs53576 (the lower anxiety in more minor allele carriers), and rs2228485 variations correlated with anxiety adjustment. Dispositional empathy is outstandingly correlated with three *OXTR* SNPs: rs2228284, rs1042778 (lower scores in carriers of the minor allele), and rs53576. Also, it was found that *OXTR* rs53576 variation was also associated with IOS scores (heterozygote carriers showed lower mean scores) and rs2228485 (Pearce et al., 2018a).

Couple empathic communication paves the way for improving better and more intimate emotional relationships. In contrast, the existence of problems with effective empathy in couples’ relationships often results in the weakening of the relationship and emotional distress (Anderson and Saunders, 2003). Schneiderman et al. (2014), regarding the correlation between the *OXTR* gene and empathic connection problems at the beginning of romantic love formation, found that having alleles with a higher risk in the *OXTR* gene can have supportive effects on the problems of establishing empathic relationships at the beginning of the romantic love formation. It was also found that individuals who have higher-risk alleles in the *OXTR* gene (rs1042778, rs2254298, rs13316193, rs2268494, and rs226849) showed less empathy for their partner’s distress, less emotional congruence, presented less social reciprocity, and paid less attention to their partners’ communication while maintaining focus on support provision (Schneiderman et al., 2014).

Sex differences in the functioning of the OT pathway are currently under active discussion. Data on the effect of sex hormones on the function of the oxytocinergic system are limited (Van Anders et al., 2011; MacDonald, 2013); however, it is known from animal studies that estrogens stimulate OT production and testosterone acts through the vasopressin pathway (Gabor et al., 2012). Women have been shown to respond to problems in couples with increase in the OT concentration, whereas men respond to vasopressin (Taylor et al., 2010). Some researchers believe that gender variances in the production and uptake of OT can principally regulate the general pathways of stress response in individuals of different sexes, from support-seeking behavior in women (tend-and-befriend behavior) to “fight or flight” strategies in men (Olff et al., 2013; Torres et al., 2018). It is known that the size of the amygdala, where numerous OXTRs are localized, is larger in men than in women. At the same time, the larger size of the amygdala is related to less prosocial behavior. It was shown that only in men, a homozygote G allele of the *OXTR* rs53576 gene was correlated to smaller amygdala size (Tost et al., 2010), poorer stress tolerance (Lucas-Thompson and Holman, 2013), and augmented sympathetic response to stress (Norman et al., 2012). At the same time, Japanese male GG of rs53576 carriers (but not females) are characterized by a higher level of confidence (Nishina et al., 2015).

DNA methylation, as an important process, has an essential role in gene expression silencing (Meloni, 2014). Some studies propose that close relationships are impacted by experiencing stressful situations (Baker et al., 2017); in this condition, DNA methylation at the *OXTR* gene can be considered an essential mechanism. In a study by Steven M. Kogan et al. (2019), the possible role of *OXTR* DNA methylation in relationship alterations was evaluated in response to adversity in childhood and socioeconomic instability. Their findings indicated that *OXTR* methylation was associated with these situations throughout a 1.5-year period (Kogan et al., 2019). In another study, Ronald L. Simons et al. (2017) suggested that the epigenetic mechanisms involved in the regulation of the oxytocin pathway may be a biological path involved in the negative cognitions central to depression.

### CD38 molecule (*CD38*)

CD38 is a transmembrane protein involved in the regulation of OT production, cell differentiation, and migration; the prominent expression of CB38 was recorded in hypothalamic neurons and lymphocytes (Jin et al., 2007; Higashida et al., 2019). *CD38* knockout mice are characterized by markedly reduced OT production and impaired social functions (Liu et al., 2008). An allele variant of rs3796863 in the *CD38* gene (4p15 chromosomal region) is associated with high OT production and socialization (Malavasi et al., 2008; Feldman et al., 2012). Makhanova et al. (2021) evaluated the association between *SNP rs3796863* and bonding-relevant cognition and relationship satisfaction over the first 3 years of marriage. They exploited data from a longitudinal study of newlywed couples to scrutinize whether variation in rs3796863 was related to relationship processes and consequences in newlywed couples. The CC genotype (vs. AC/AA) correlated with increased partner appreciation, trust, and forgiveness. Furthermore, contributors with the CC genotype (vs. AC/AA) had higher levels of relationship satisfaction. Finally, considering the higher level of satisfaction among individuals with the CC genotype (*versus* AC/AA) after 3 years of marriage and the initiation of romantic relationships, they suggested that the rs3796863 variant may have persistent effects. In another study, Gentiana Sadikaj et al. (2020) showed that *CD38* rs3796863 was associated with an individual’s interpersonal communication behavior, such as expressing love to a romantic and emotional partner (a person with the CC genotype has a more cooperative behavior than a person who has the A allele). In conclusion, *CD38* rs3796863 is related to relationship adjustment so that a person with the CC genotype has higher levels of global relationship adjustment than those with other genotypes (Sadikaj et al., 2020).

### Arginine vasopressin receptor 1A (*AVPR1A*)

Two related neuropeptides, the arginine vasopressin and oxytocin hormone, are well-conserved during evolution and have been shown to play a role in various social behaviors, memory, and learning (Van Kesteren et al., 1995; Donaldson and Young, 2008; Veenema and Neumann, 2008). The *AVPR1A* gene encodes a receptor that mediates the effects of the AVP hormone in the central nervous system (brain) (Wassink et al., 2004; Fink et al., 2007). It has been exposed that *AVPR1A* plays a role in regulating and modulating social cognitive processes and behaviors such as adaptation, social bonds, and altruism (Donaldson and Young, 2008).

The study of Pearce et al. (2018a) showed that *AVPR1A* variation could have significant effects only on disposition, dyadic, and network-level relationships. Also, in a previous study, they found that repeat length polymorphisms in the *AVPR1A* gene can have a meaningful relationship with differences in sexual behavior (Prichard et al., 2007; Walum et al., 2008; Pearce et al., 2018a). In a study of twins and their romantic relationships with their partners, Walum et al. (2008) showed that *AVPR1a* rs3 was associated with higher rates of partner bonding, fewer relationship difficulties, greater obligation, and better quality of their romantic relationship (only in men). Another study revealed that this variant correlated with a higher level of sexual satisfaction and sexual activity frequency (Acevedo et al., 2019). Furthermore, *AVPR1a* rs3 can play a role in complex social cognitive processes like empathy, emotional facial reaction, and altruism (Volbrecht et al., 2007; Meyer-Lindenberg, 2008; Brunnlieb et al., 2016). Interestingly, it has been proposed that there is a significant interaction with this polymorphism and romantic love continuation in the right VTA, the PAG, the posterior hippocampus, the occipital cortex, and the superior temporal gyrus (STG; a critical region for the prize, adaptation, memory, and visual and sensory processing) (Schultz et al., 2003; Nagy et al., 2012; Acevedo et al., 2020). These regions are typically seen in the background of long-term romantic love (Acevedo et al., 2012).

### Serotonin-related signaling pathway

Serotonin [5-hydroxytryptamine (5-HT)], a neurotransmitter, has many physiological roles, such as regulating the transcription of many genes and the activity of neurotrophic factors and steroids. Therefore, this neurotransmitter can profoundly affect various brain activities, such as cognitive control, learning and sensory processing, regulation of emotions and feelings, autonomic nervous system responses, memory and sleep, and motor function (Palacios, 2016).

5HT is released from the end of the axons of presynaptic neurons into the synaptic space to bind to their receptors. These receptors are divided into seven subfamilies based on their structural features and conserved domain and their signaling pathway (ionotropic receptor 5-HT3 and G protein-coupled receptors including 5-HT1, 5-HT2, 5-HT5, and 5-HT4/6/7) (Millan et al., 2008; Palacios, 2016) (Figure 3). The number of receptors is strongly influenced by alternative splicing and the RNA editing process. For example, there are 10 splicing variants for the 5-HT4 receptor. 5-HT1 and 5-HT5A receptors negatively inhibit adenylyl cyclase (AC), which ultimately inhibits cAMP repletion. Also, the 5-HT1 receptor activates the phospholipase C (PLC), inositol-1,4,5-triphosphate (IP3), and diacylglycerol (DAG) signaling pathways. Activation of this pathway causes the release of calcium from the endoplasmic reticulum and the activation of protein kinase C (PKC) (calcium/calmodulin-dependent kinases) (Figure 3). Presynaptic localization of 5-HT1B is assumed to repress excessive 5-HT secretion (Nichols and Sanders-Bush, 2001; Millan et al., 2008; Masson et al., 2012).

### Solute carrier family 6 member 4 (*SLC6A4*)

*5-HTTLPR* (*5-HTT*-related polymorphic) is a polymorphic region in the promoter of the serotonin transporter gene (*SLC6A4*). The chromosomal location of *5-HTTLPR* is 17p13, and its genomic region includes a single promoter and 14 exons. The genomic region of the promoter of the gene (*5-HTTLPR*) contains two alleles: the short allele (*s*) (12 copies) and the long allele (*l*) (14 copies). The difference between these two alleles is the number of copies of a 22-bp repetitive sequence. However, a significant proportion of African–Americans have a longer 16-copy variant. The length of the repeat is related to the amount of 5HTT production so that the “*s*” variant is associated with less production and a decrease in the efficiency of 5-HT uptake. The consequences of this reduction are depression or development of impulsive aggression, which are essentially related to the serotonin signaling pathway (Carver et al., 2008; Vijayendran et al., 2012). It has been shown that *5-HTT* variation can affect marital interactions toward improvement or worsening (Schoebi et al., 2012). In response to marital impulses (whether positive or negative), the level of marital satisfaction in *s* allele carriers is more affected. These individuals are strongly influenced by positive or negative feelings and emotions (Haase et al., 2013). Similarly, Man-Kit Lei et al. (2016) showed that the “*s*” allele could be considered a “susceptibility” allele in the process of the impact of underlying stress on romantic relationship satisfaction; thus, *5-HTTLPR* variation can increase the sensitivity to environmental influences of stressful factors on a romantic relationship (Lei et al., 2016). In another study, Starr and Hammen (2016) proposed that the “*s*” allele of *5-HTTLPR* has a strong linear relationship with the degree of romantic relationships and depressive symptoms. The “*s*” allele is associated with a higher susceptibility to depression after a romantic relationship, which can be provoked by chronic stress and exacerbated by higher levels of family conflicts. Siyang Luo et al. (Starr and Hammen, 2016) examined the theory that *5-HTTLPR* is associated with individuals’ romantic relationship satisfaction (RRS). By using the fMRI (functional MRI) technique and comparison of homozygote (*s/s*) and (*l/l*) individuals throughout a Cyberball game that led to deprivation of social actions, they also investigated the effect of *5-HTTLPR* on neural activity that could be related to RRS. Compared to *s/s* homozygotes, *l/l* homozygotes had higher levels of RRS, lower levels of social interaction anxiety, stronger activity in the right ventral prefrontal cortex (RVPFC), and stronger functional connectivity between the dorsal and rostral ACC at game withdrawal time. It was shown that RVPFC activity is involved in the association of *5-HTTLPR* with RRS. In contrast, the correlation of *5-HTTLPR* with social interaction anxiety is moderated by both dorso-rostral ACC connectivity and RVPFC activity (Starr and Hammen, 2016).

### 5-Hydroxytryptamine receptor 1A & 2A (*5-HTR1A* and *5-HTR2A*)

Along with the serotonin transporter, the serotonin receptor regulates serotonin levels in the brain (Le François et al., 2008; Trueta and Cercós, 2012). The 5-hydroxytryptamine 1A receptor is one of the key receptors expressed in pre- and post-synaptic neurons of mammalian brain (Drago et al., 2008); their stimulation on the dendritic terminals of neurons (in the cortex and hippocampus) creates a negative feedback loop for secretion of serotonin (Sprouse and Aghajanian, 1987). 5-HT1A receptor expression is regulated by the *5-HT1A* gene. A single-nucleotide polymorphism (C-1019G) in this gene regulates the expression of 5-HT1A receptors (Lemonde et al., 2003; Albert and Lemonde, 2004; Le François et al., 2008). The G allele (compared to the C allele) is associated with reduced levels of serotonin in the synaptic space (Drago et al., 2008; Le François et al., 2008; Trueta and Cercós, 2012) and a higher risk for depression (Lemonde et al., 2003; Albert and Lemonde, 2004; Czesak et al., 2012). In another study, it was shown that GG homozygotes (compared to CC homozygotes) had higher scores on the Toronto alexithymia scale (TAS-20). Also, it was found that people with the CG/GG genotype (compared to C/C genotype carriers) feel less comfortable and intimate with being in a close relationship (Gong et al., 2014). Additionally, the results of Jinting Liu’s (Gong et al., 2014) study regarding the effect of this polymorphism on romantic relationships confirmed that individuals with the CG/GG genotype (compared to C/C genotype carriers) prefer to be single. Indeed, individuals with the CG/GG genotype are more likely to have a higher degree of neuroticism (Strobel et al., 2003) and may also have a range of psychiatric problems such as major depression (Lemonde et al., 2003; Kishi et al., 2013) and borderline personality disorder (Joyce et al., 2014). Considering that neuroticism can make it difficult to form a close relationship, especially romantic relationships, and affect its quality (Lehnart and Neyer, 2006; Assad et al., 2007), the presence of the G allele may increase the chances of finding an emotional partner or affect maintaining a romantic relationship.

In humans, “loving styles” and variations in *DRD2* and *HTR2A* genes are somehow linked together (Miller et al., 1999), so it can be assumed that the *HTR1A* variation is associated with the formation of romantic relationships (Emanuele et al., 2007) and *OPRM1* and *HTR2A* with differences in mate selection success differentially across sexes in “speed-dating” circumstances (Wu et al., 2016). Pearce et al. (2017) indicated that *HTR1A* gene variation was associated with a more comprehensive social network (network size). Furthermore, in another study, they showed that there is a correlation between *HTR2A* variation and individual differences in social interaction (including others in the self-scale (IOS) and network size) (Pearce et al., 2017). Additionally, in another research, they showed that *HTR2A* variation was associated with SOI-R ( Sociosexual Orientation Index-Revised) scores (Pearce et al., 2018a). Interestingly, it has been identified that an *OPRM1* SNP predicts speed-dating success in women, while the *HTR2A* variant is associated with dating success in men (Wu et al., 2016).

### Dopamine- and catecholamine-related signaling pathway

The dopaminergic system is a fundamental pathway for controlling voluntary movements, control of pleasure circuitry, regulation of mood, attention, cognitive functions, sleep, appetite, sense of smell, vision, and erections (Beaulieu et al., 2015). Dysfunction of the dopaminergic system is associated with a variety of neuropsychiatric disorders, including schizophrenia, Parkinson’s disease, and addictive, anxiety-depressive, obsessive-compulsive, attention deficit hyperactivity, and eating disorders (Asl et al., 2019; Klein et al., 2019; Yu et al., 2022).

Five types of dopamine receptors in humans are encoded by the *DRD1*, *DRD2*, *DRD3*, *DRD4*, and *DRD5* genes. Dopamine receptors are a member of the of G protein-coupled receptor superfamily, and they include two subfamilies; D1-like (comprising D1 and D5; stimulating secondary messenger and binding to canonical Gs/olf proteins) and D2-like (comprising D2, D3, and D4; inhibiting secondary messenger and binding to Gi/Go proteins) (Figure 3) (Neve et al., 2004; Beaulieu and Gainetdinov, 2011).

The *COMT* gene encodes the enzyme catechol-O-methyltransferase (COMT), which cleaves dopamine in the prefrontal cortex. The rare “A” allele alters the structure of the resulting enzyme so that its activity is only 25% of that of the wild type. As a result, carriers of the A allele have more dopamine in the prefrontal cortex compared to a carrier of the wild-type G allele, which may cause many neuropsychological associations (Tunbridge and Harrison, 2010).

Studies have shown that *ANKK1* (ankyrin repeat and kinase domain containing 1) gene variants may be involved in the formation of addictive behavior, possibly by influencing the development and function of the dopaminergic system (Koeneke et al., 2020). A recent study showed a likely functional link between *ANKK1* and *DRD2* (Leggieri et al., 2022).

Little is known about the role of the dopaminergic signaling pathway in establishing and maintaining romantic and emotional relationships in humans. However, animal studies provide strong evidence for such a relationship (Acevedo et al., 2012). For example, Young and Wang considered a special worth for the function of brain structures that implement the production and reception of dopamine for pair bonding, revealing sex differences in the relationship at the same time (Young and Wang, 2004). Subsequently, these findings were partially confirmed in a neuroimaging study in humans: the passionate stage of romantic love is accompanied by a pronounced activation of the dopaminergic system in two regions, the medial orbitofrontal cortex and medial prefrontal cortex (Takahashi et al., 2015). Accumulated empirical evidence has led to the “dopamine hypothesis of romantic love,” according to which romantic love is based on a motivational drive resulting from “natural addiction,” given that the role of dopamine in the formation of pathological addictions is well-established (Fisher et al., 2006; Frascella et al., 2010; Fisher et al., 2016; Wang et al., 2020). According to our search strategy, we found six studies on the link between dopamine receptors and couple adjustment in the context of romantic relationships. Two articles investigated the role of the *DRD4* gene, and four articles investigated the role of *DRD1* and *DRD2*.

A series of articles by Pearce et al. (2017), Pearce et al. (2018a), and Pearce et al. (2018b) showed that the *DRD1* rs265981 and *DRD2* rs1076560 polymorphisms are associated with reduced receptor ligand-binding capacity (Bertolino et al., 2010) and are prone to enhance social connections in seeking a partner and a higher sociosexuality index, suggesting higher levels of short-term romantic relationships. The indicated associative relationship was observed in both pilot (Pearce et al., 2017) and replicative (Pearce et al., 2018a) studies, increasing the evidentiary strength of the authors’ findings. At the same time, the authors showed that *DRD2* rs4648317 is not associated with sociosexual traits and relationship quality (Pearce et al., 2018b). In the mentioned study, no relationship was found between positive parental involvement in adolescents’ lives, strength of romantic relationships, and Taq1 A polymorphism in *ANKK1*/*DRD2* rs1800497 (Masarik et al., 2014). However, an association of *ANKK1*/*DRD2* rs1800497 with an index of sociosexuality (Pearce et al., 2017; Pearce et al., 2018a) was found.

Acevedo et al. (2020) showed that the presence and maintenance of romantic involvement over time positively correlated with numerous 7R alleles of the *DRD4* gene (Acevedo et al., 2020). Similar results obtained by Masarik et al. (2014) show the relationship between positive parental involvement in adolescents’ lives and the *DRD4*-7R polymorphism was statistically significant (in a regression model, ß = .447, *p* < .05), which subsequently has a positive effect on romantic relationships. The 7R allele is thought to be associated with a reduced ability of DRD4 to bind dopamine and a greater need for risk-taking behaviors, more diverse sexual behaviors, a greater desire for children early in love relationships, and novelty-seeking (He et al., 2018; Acevedo et al., 2020). It seems possible that individuals with a higher number of 7R alleles of the *DRD4* gene tend to be more romantically intense in short-term relationships but less able to maintain relationships in the longer term (Minkov and Bond, 2015).

Two studies assessed the associative relationship of *COMT* gene polymorphisms. In the study mentioned previously, carriers of the *COMT* rs4680 allele “A” were found to have higher levels of romantic love maintenance and response to a partner (Acevedo et al., 2020). As previously indicated, carriers of the *COMT* rs4680 allele “A” were characterized by higher levels of dopamine in the prefrontal cortex. Masarik et al. (2014) found no relationship of sufficient significance between *COMT* rs4680 and the studied parameters of parental involvement in adolescents and romantic relationships in adulthood.

Thus, the results of the studies included in the review provide moderate evidence for the involvement of genetically programmed features of the dopaminergic system in the establishment and maintenance of romantic relationships. Genotypes associated with low dopamine receptor binding capacity (*DRD1* rs265981, *DRD2* rs1076560, *ANKK1*/*DRD2* rs1800497, and *DRD4*-7R) may contribute to a stronger romantic relationship, at least in the short term (for example, in the initial period of the marriage). Characteristically, these same genotypes are associated with various types of pathological addiction, which, in a way, confirms the “dopamine hypothesis of romantic love” in terms of the molecular–genetic basis. However, to confirm such associations, more extensive studies with larger numbers of participants, a prospective design, and consideration of gender differences are required. Studies of *COMT* gene involvement are presented as single studies and do not allow for drawing unequivocal conclusions.

## Other genes related to romantic relationships

### Major histocompatibility complex (MHC)

The major histocompatibility complex (MHC) or human leukocyte antigen gene set is located on human chromosome 6, which encodes cell surface markers and has critical roles in cell immunity function (Migalska et al., 2019). MHC loci are highly polymorphic (Bertaina and Andreani, 2018), and this variation is pivotal owing to the diversity of the major histocompatibility complex (MHC) and is important for health and fitness. MHC genotypes can predict the quality or compatibility of an individual as a competitor, ally, or partner, which might be because MHC products can affect the components of the body's secretions. In addition, human body odor indicates the MHC composition and can affect spouse identification and selection (Havlíček et al., 2020). Christine E. and colleagues show that MHC molecules can also play a role in mate selection and sexual satisfaction. It has been reported that mate choice in vertebrates can be related to the association between MHC and smell; for example, mice, by smell, choose a mate that has a different MHC genotype (Garver-Apgar et al., 2006). The evidence for this preference among humans is generally positive but mixed. Three of four studies of women who are normally ovulating demonstrated a preference for positive scents from opposite-sex individuals with dissimilar MHC genotypes. Based on the results of studies, MHC sharing adversely affects women’s sexual responsiveness and sexual satisfaction with partners. Moreover, women tend to have extra-pair partners (solely in their present relationship) and are more attracted to extra-pair men apart from their main partner, which is particularly aggravated during the fertile days of the menstrual cycle (Garver-Apgar et al., 2006; Havlíček et al., 2020). Current studies from J. Kromer and colleagues have shown that immunological compatibility is important in a relation, relationship and sexual satisfaction, and the desire to have a child. Therefore, in a romantic relationship, if the emotional partners have different HLA, the level of relationship satisfaction increases. This effect was evident only with the HLA class I allele but not with the class II allele (Kromer et al., 2016).

### Y-DNA haplogroup D-M55

It has been shown that spermatogenic failure is associated with Y-DNA haplogroup D-M55, and sperm count is negatively associated with body mass index (BMI). It may be indirectly concluded that BMI and Y-DNA haplogroup D-M55 may be related. Considering the relationship between BMI and psychological or social parameters, it was suggested that they might be related to haplogroup D-M55 (Sermondade et al., 2012; Sato et al., 2013; Mchiza et al., 2019). Matsunaga et al. (2021) studied the psychological (behavioral indicators, number of near companions, and feelings of happiness and loneliness) and physiological (BMI) parameters’ impacts of the haplogroup D-M55 in Japanese people. The results showed that men with the haplogroup D-M55 genotype (compared to non-carrier men or women) had a higher body mass index and a greater number of near companions. In contrast, they did not show a significant difference in the level of happiness or loneliness. Considering that *DRD2* rs1800497 is associated with BMI and individual behavior and communication, it was determined that they are related to each other through gene–gene interactions.

### Gamma-aminobutyric acid type A receptor subunit alpha-2 (*GABRA2*)

GABA is a neurotransmitter that functions as an inhibitory neurotransmitter in the central nervous system, reducing neuronal excitability (Edenberg et al., 2004). GABA-A receptors can be found all over the brain. GABA-A receptors are composed of at least 16 different subunits. GABRA2 is a GABA heteropentameric receptor component that is a ligand-gated chloride channel. GABRA2 is involved in the formation of functional inhibitory GABAergic synapses. Because GABRA2 affects GABA, there is a good possibility that variations in this gene alter vulnerability to social–environmental events. The role of the *GABRA2* minor (G) allele in adulthood behavior is supported by replicated previous findings (Covault et al., 2004; Edenberg et al., 2004). Several studies have found that being subjected to harsh parenting as a child increases the likelihood of developing hostility toward adult marital partners. The gene GABRA2 influences this association (Carr and VanDeusen, 2004; Black et al., 2010). Ronald L. Simons et al. (2013) studied how GABRA2 gene variations interact with parental behavior in the way the differential susceptibility perspective predicts. They found that individuals with the *GABRA2* minor (G) allele genotype (compared to major allele carriers) were more verbally and physically aggressive toward their romantic partner during a romantic relationship if they were exposed to parental violence as children. However, if they have been subjected to the supportive behavior of their parents, they will show a lower level of aggression. GABRA2 may also be a plasticity gene according to their findings. Carriers of minor alleles are likely sensitive to many problematic behaviors in reaction to bad circumstances, not just relationship aggression. However, when environmental conditions are perfect, they are likely to succeed in comparison to other genotypes (Simons et al., 2013).

### μ-Opioid receptor gene (*OPRM1*)

The brain opioid theory of social attachment suggests that changes in the activity of opioids created in the endogenous pathways may make a person feel closer and more intimate in social relationships and experience less suffering caused by loneliness and social separation (Panksepp, 2004). One study found that when women recalled the memory of the loss of a loved one, they showed reduced μ-opioid receptor-mediated neurotransmission. It also observed that inactivation of the μ-opioid receptor was straightly related to self-reported negative affective experiences (Zubieta et al., 2003). One of the most studied variants is *OPRM1* A118G (rs1799971) (Bond et al., 1998), the genotype with the G allele that has been associated with decreased gene expression and consequently lower receptor protein translation (Zhang et al., 2005), followed by reduced (brain region-dependent) opioid signaling efficiency (Oertel et al., 2009). Tchalova et al. (2021) showed that G allele carriers (compared to AA homozygotes) experience more insecurity during an emotional relationship mixed with violence. They also proposed that men carrying the G allele genotype (compared to men homozygous for the A allele) experience less security feeling in all occurrences of their relationships, whereas the partners of these men also experience similar feelings (Tchalova et al., 2021). In research by Pearce et al. (2017), Pearce et al. (2018a), and Pearce et al. (2018b), they showed that *OPRM1* variants correlated with anxious attachment, empathy, EQ (emotional intelligence), quality of the emotional and romantic relationships, sexual tendency or activities, and the ability to empathize (Pearce et al., 2017; Pearce et al., 2018a; Pearce et al., 2018b). Dopamine and endorphin signaling pathways may be related and interact because *OPRM1* and *DRD2* gene variants are inevitably associated with community integrity (Devine et al., 1993; Pecina et al., 2015).

## Discussion

To our knowledge, this is one of the few studies in human behavioral genetics considering the impact of single-nucleotide variations on the individual’s conduct in close relationships. It should be noted that the evidence for genetic influences on romantic relationships does not suggest the absolute, immutable effect of genes on relationships (Whisman and South, 2017). Research investigating gene–environment correlations and gene–environment interactions that take into account psychopathic states, social welfare, and physical healthiness could have consequential outcomes on romantic relationships (Pearce et al., 2018a). It can be considered that the intricate interactions between various polymorphisms in different genes and the collection of environmental features such as educational strategy, social stressors, economic situations, and cultural conditions can involve in the determination of an individual’s behavior in a romantic relationship (Whisman and South, 2017; South, 2021). For example, low income or incoming stress is considered a significant threat to marital quality and stability (Lichter and Carmalt, 2009). It has been found that the religiosity of couples has a positive effect on marital satisfaction and marriage survival (Marks, 2005). Collectivistic and individualistic cultures have different effects on marriage satisfaction in people. For example, fulfilling familial responsibility may enhance marital satisfaction in traditional Chinese marriage (Wang, 1994), whereas fulfilling the hedonistic goals of partners seems to be beneficial for marital satisfaction in Western countries (Lalonde et al., 2004; Sorokowski et al., 2017).

The genotype, environmental background, and psychological state of the partner play an effective role in a romantic relationship and mutual satisfaction (Harden, 2012; Schneiderman et al., 2014; Shpigelman and Vorobioff, 2021). For instance, it has been found that an individual’s outcome in a romantic relationship is related to the rs3796863 SNP in the *CD38* gene of the partner. Data revealed that the CD38 rs3796863 genotype can display a “partner effects,” which means the partner’s CD38 rs3796863 genotype is associated with the individual’s satisfaction in romantic love (Sadikaj et al., 2020). In other words, the outcome of the relationship depended as much on her genotype as on her partner’s genotype (Sadikaj et al., 2020). Moreover, it has been reported that individuals who had a spouse with the GG genotype in OXTR rs53576 had higher satisfaction in marriage (Monin et al., 2019). In this way, to evaluate the impact of single-nucleotide polymorphisms on romantic relationships, the partner’s genome has an important role. Thus, it should be examined (Monin et al., 2019).

There is sufficient empirical support for the critical role of the dopaminergic system in the formation and maintenance of romantic relationships. For example, the receptor variant *DRD4*-7R is associated with features of sexual behavior, the desire for more diverse sexual experiences, higher levels of promiscuity, and a higher intensity of romantic relationships in the short term, such as the initial period of relationship formation (Halley et al., 2016; He et al., 2018; Acevedo et al., 2020). Regarding the dopamine- and catecholamine-related signaling pathway, the studies analyzed in this review focused on three dopamine receptor genes (*DRD1*, *DRD2*, and *DRD4*) and the catechol-O-methyltransferase (*COMT*) gene. The analysis showed variants that reduce the binding capacity of the receptor to dopamine (*DRD1* rs265981, *DRD2* rs1076560, *ANKK1*/*DRD2* rs1800497, and *DRD4*-7R) could affect the romantic relationship in couples. In contrast, for the COMT gene, the results were inconsistent.

However, the described genomic correlations require further studies. For example, the need to examine the role of genes in emotional relationships by considering the differences between the two genders and designing a long-term study to evaluate the role of identified correlations not only in the short-term period of relationship formation but also in the long-term maintenance of the relationship seems to be important in future studies. Cimbalo and Novell (1993) reported sex differences in romantic love attitudes among college students. They concluded that in relationships, women would consider marriage and family, traditional romantic behavior, routine activities, and religion desirable, whereas men would consider sexual behavior, aberrant sex, and drugs more desirable

A significant amount of research has shown critical behavioral differences between the early and later stages of a romantic relationship (Eastwick and Finkel, 2008; Acevedo and Aron, 2009). In this regard, it is reasonable to assume that carriers of *DRD4*-7R (Acevedo et al., 2020), *DRD1* rs265981, and *DRD2* rs1076560 (Pearce et al., 2018a) will have a greater propensity to seek new partners after a high-intensity “honeymoon” relationship, which will affect the quality of the established marriage. In addition, limited findings on the involvement of the dopaminergic system in shaping romantic relationships may also be due to the ethnic and sociocultural heterogeneity of the samples (Pearce et al., 2018a).

### Implications

The results of this review may have practical applications, suggesting that educational and therapeutic programs in early marriage can focus on strengthening the romantic side of the relationship to increase marital satisfaction, as indicated by some researchers (Acevedo and Aron, 2009). For example, findings (Acevedo et al., 2012; Acevedo et al., 2020) indicate that romantic love is associated with greater involvement, less alternative partner attraction, and long-term marital satisfaction and is associated with a neurogenetic basis for sustained reward, which may be a target of psychological techniques. In addition, as the included studies suggest, some genetic variants positively correlated with the strength of romantic relationships (e.g., *DRD1* rs265981 and *DRD2* rs1076560) (Pearce et al., 2018a) are also associated with enhanced social connections, which may be used to shape therapeutic programs.

Painful/rejected romantic infatuation can also have negative consequences, including heartbreak, which can provoke family violence, depression, and even suicide. The neurobiological similarities of romantic desire with other substance and non-substance addictive disorders (including those confirmed by the cited neurogenetic studies in this review) may help use already known drug and non-drug addiction treatment techniques to overcome the possible negative consequences of painful/rejected romantic infatuation (Fisher et al., 2016; Zou et al., 2016). In particular, group techniques that positively influence the reward system and exercise that increases dopamine secretion can be used. The neurogenetic predisposition to intense romantic infatuation and chemical addiction shown in this review may have implications for forming groups at risk for substance abuse as a reaction to rejected love.

The neurogenetic correlates described in this review can be used to find new drug therapies for pathologic jealousy as an extreme manifestation of intense romantic infatuation. In particular, the use of intranasal oxytocin (Samad et al., 2019; Zheng and Kendrick, 2021) and antipsychotics with dopaminergic activity (Samad et al., 2019) has already been proposed for this purpose.

The concept of “social connection” can be defined as the feeling that you belong to a social relationship and generally feel close to other people. Sociality is a core psychological need for humans. The tendency to associate in a social network is embedded in our biology and evolutionary history. The size and quality of social networks are increasingly being linked to mental and physical health, happiness, longevity, faster recovery from illness, and lower odds of engaging in anti-social behavior or experiencing psychopathology (Schindler and Sack, 2015; Delvecchio et al., 2016). For example, according to findings, across all three types of samples (a healthy Caucasian sample, a subclinical sample (Caucasian individuals with histories of mental illness), and a non-White sample), dopamine-related gene variation was linked to engagement in the large network beyond the dyadic bonds (in romantic relationships). *DRD1* showed significant relationships with both personal network size and intimacy to the local community scores in the non-Caucasian sample; in contrast, *DRD2* did so in the other two samples (Pearce et al., 2018a). Polymorphisms in the oxytocin receptor (*OXTR*) gene can influence the social skills that are important for building and maintaining social connections. *OXTR* rs53576 is an intensively examined polymorphism in the oxytocin receptor (*OXTR*) gene in relation to individual differences in social cognition. It has been indicated that the scores of interpersonal adaptability and dispositional forgiveness are increased in individuals who have the G allele of *OXTR* rs53576 (Aspé-Sánchez et al., 2015). Moreover, the G allele of *OXTR* rs53576 is indeed associated with better empathic ability (Gong et al., 2017). Another polymorphism, *OXTR* rs2254298, can affect limbic system structure and function (Tost et al., 2011), and C carriers of this polymorphism displayed more cognitive empathy than those with the TT genotype (Wu et al., 2012). An association between emotional empathy and *OXTR* polymorphism at the rs237887 SNP (with A allele subjects displaying higher than those with the G allele) and the rs4686302 SNP (with T allele subjects displaying higher than those with the C allele) was reported, whereas cognitive empathy and the ability to understand another’s perspective or mental situation showed associations with SNP polymorphisms rs2268491 (with T/T and C/T genotypes displayed higher than those of the C/C) and rs2254298 (with CC and C/T genotypes displayed higher than those of T/T genotypes) (Wu et al., 2012). Studying these polymorphisms in the context of romantic relationships may pave the way to shaping therapeutic programs.

Personality traits such as extraversion and neuroticism are often associated with diverse areas of sexual activity and sexual healthiness. Data regarding the sexual behavior of patients affected by major psychiatric disorders reported various derangements in sexual behavior and sexual performance (Kurpisz et al., 2016; Decaro et al., 2021). Psychiatric patients rarely speak of their sexual life spontaneously. It has been found that there is a positive link between the Big Five personality dimensions and sexual function, risky sexual behavior, sexual disorders, sexual satisfaction, emotional experiences in sex, attitude toward sex, and sexual unfaithfulness (Kurpisz et al., 2016). Lower levels of sexual performance and physical and emotional satisfaction are shown in schizophrenia patients (Long et al., 2022; Ludwig et al., 2022). Research on finding the association between affecting SNPs on the development of pathobiology of psychiatric disorders such as schizophrenia, depression, and bipolar disorder and formation and maintenance of couple romantic relationships could be helpful to pave the way to reveal the role of these variants in the sexual and emotional life of the population.

### Limitations and future research

The studies included in the review tend to have very small sample sizes and require verification in replicative and GWAS projects. At the same time, highly evidence-based GWAS projects require very large sample sizes and significant funding (Landefeld et al., 2018). Other important genetic variants could also be investigated in future studies. For example, the 5-HTTLPR VNTR of the serotonin transporter gene is involved in the degree of risk tolerance in marital relationships and may influence the longevity of romantic relationships in a couple (Minkov and Bond, 2015). Also, given the available neurobiological background, opioid receptors, testosterone, and cortisol genes may be investigated (Ponzi and Dandy, 2019; Meyer and Sledge, 2020).

It is essential to consider the effect of cultural conditions in society besides the genetic investigations on marriage satisfaction. Cultural values and beliefs affect the individuals’ self-construal and knowledge of love and relationship (Dion and Dion, 1993). Individualism and collectivism are cultural values that influence the relationship between the person and society (Dion and Dion, 1993). Romantic love and psychological intimacy play more important roles in marriages in individualistic cultures (compared with collectivistic cultures). In collectivistic cultures, relationship with other family members (e.g., parents, siblings, and in-laws) has an important position (Wong and Goodwin, 2009; Chiu and Hong, 2013; Carollo et al., 2021). Cross-cultural studies of love and intimacy have reported differences between individualistic and collectivistic cultures. For instance, the husband’s income is associated with marital satisfaction in collectivistic cultures but not in individualistic culture (Kamo, 1993). In contrast, the same level of education increases marriage satisfaction in individualistic cultures (Groot et al., 2002).

The ancestry of the target population as a limitation must be considered. Population stratification refers to allele frequency differences between case and control groups due to systematic differences in ancestry (rather than the association of genes with disease) (Freedman et al., 2004). Population stratification is one source of false-positive associations (Knowler et al., 1988; Kittles et al., 2002). It has been proposed that genotyping several unrelated genetic markers may reduce the number of false-positive associations caused by stratification. However, there has been much debate but limited data about the impact of population stratification on case-control association studies (Thomas and Witte, 2002; Wacholder et al., 2002; Cardon and Palmer, 2003; Hoggart et al., 2003). It has been proposed that the stratification effects may be successfully ignored by precisely matching patients and controls based on their own ancestry and geographical origin (Wacholder et al., 2002). Thus, due to the allele frequency differences among dissimilar populations, the establishment of population-specific studies is needed, and these findings may not be generalizable to other populations.

Future research should focus more on assessing gene–environment correlations and gene-by-environment (GxE) interactions in the context of romantic relationships (Whisman and South, 2017). Evaluating genetic factors on relationship aspects at times of important changes in a couple’s life, such as offspring emergence, career development, and possible personal and financial micro-crises, also seems extremely important. The formation of samples with established homogeneity according to age, ethnicity, social, cultural, and financial status is also of undeniable importance (Karney and Bradbury, 1995). As mentioned previously, ethnicity is very important for assessing the role of the dopaminergic system (Pearce et al., 2018a). Young and Wang (2004) emphasized the critical role of the function of brain structures that implement neurotransmitter production and reception for pair bonding, while emphasizing the sex differences simultaneously. At the same time, overly restricted samples invariably lead to limited conclusions that are valid only for narrow social groups (Lavner and Bradbury, 2010). Thus, a larger sample size with the ability to stratify by likely trajectories of couple relationships, many other intervening factors, and a longitudinal project design is critical for future research. In addition, the articles cited in the review deal exclusively with heterosexual relationships, and research on homosexual couples may also be of interest in this aspect.

## Conclusion

In conclusion, romantic love can change the development, dimensions, stability, and feeling of satisfaction in a relationship. Due to the significant advances in biological sciences, especially genetics, there is a need to understand what biological mechanisms are involved in the formation and maintenance of romantic love. In this systematic review, we investigated genetic and epigenetic factors that can influence couple adjustment, romantic love formation, and maintenance over time. Study results show that romantic love can be regulated by processes that are associated with individual genetics in response to rewards, emotions, etc. Findings suggest that genetic polymorphisms mediate variability in behaviors associated with the maintenance of romantic love and pair bonding during marriage. These findings about the genetic variations involved in romantic love can be valuable in couple therapy and counseling for forming and maintaining a romantic relationship.

## Data Availability

The datasets presented in this study can be found in online repositories. The names of the repository/repositories and accession number(s) can be found in the article/supplementary material.
